# Quantitative analysis of the macula with optical coherence tomography angiography in normal Japanese subjects: The Taiwa Study

**DOI:** 10.1038/s41598-019-45336-3

**Published:** 2019-06-20

**Authors:** Risa Sato, Hiroshi Kunikata, Toshifumi Asano, Naoko Aizawa, Naoki Kiyota, Yukihiro Shiga, Koji M. Nishiguchi, Keiichi Kato, Toru Nakazawa

**Affiliations:** 1 Department of Ophthalmology, Tohoku University Graduate School of Medicine, Sendai, Japan; 2 Department of Retinal Disease Control, Tohoku University Graduate School of Medicine, Sendai, Japan; 3 Kato Eye Clinic, Taiwa-cho, Kurokawa-gun, Miyagi, Japan; 4 Department of Advanced Ophthalmic Medicine, Tohoku University Graduate School of Medicine, Sendai, Japan; 5 Department of Ophthalmic Imaging and Information Analytics, Tohoku University Graduate School of Medicine, Sendai, Japan

**Keywords:** Structural biology, Diagnostic markers

## Abstract

This study evaluated age-related changes in the superficial and deep retinal capillary plexus (SCP and DCP, respectively) and in the foveal avascular zone (FAZ). SCP and DCP perfusion density (PD) were measured in optical coherence tomography angiography (OCTA) macular scans of 145 eyes of 145 healthy Japanese subjects, and findings were compared with SCP FAZ and clinical data. We found that age was negatively correlated with SCP and DCP PD (r = −0.17, *P* = 0.04 and r = −0.20, *P* = 0.02, respectively) and positively correlated with FAZ area (r = 0.18, *P* = 0.03). SCP and DCP PD were correlated with each other (r = 0.67, *P* < 0.001). FAZ area was negatively correlated with SCP PD, DCP PD and central macular thickness (CMT) (r = −0.18, *P* = 0.03; r = −0.25, *P* < 0.01; and r = −0.39, *P* < 0.001, respectively). FAZ was larger and CMT was lower (*P* = 0.01 and *P* < 0.001, respectively) in women than men. SCP and DCP PD were positively correlated with estimated glomerular filtration rate (r = 0.17, *P* = 0.03 and r = 0.24, *P* < 0.01, respectively). Multiple regression analysis confirmed that age independently affected DCP PD and FAZ (*P* = 0.02 and *P* < 0.01, respectively) and that CMT independently affected FAZ area (*P* < 0.001). Thus, normal subjects showed age-related decreases in macular PD and renal function. FAZ and CMT were related, suggesting that age-related changes in macular thickness also affect capillary vasculature.

## Introduction

Information on changes in the macular capillary plexus is important for a better understanding of macular disease. Particularly, a more precise evaluation of the effect of age on capillary vascularity in normal subjects might allow a more accurate analysis of disease states. Here, we used optical coherence tomography angiography (OCTA), a novel extension of the commonly used OCT technique, to investigate the macula in healthy Japanese subjects and obtain normative data.

OCTA is based on motion contrast imaging of high-resolution volumetric blood flow data. This allows the generation of angiographic image slices at a variety of depths, as well as non-invasive 3-dimensional vascular mapping of the ocular microcirculation^1–3^. A key enabling technology for OCTA has been the split-spectrum amplitude decorrelation angiography (SSADA) algorithm, which reduces noise during flow detection. This allows OCTA to produce high-quality images of the retinal vasculature^4–6^. Recently, OCTA has been evaluated in clinical studies, and has proven particularly useful in studies of retinal diseases^7–11^.

Usefully, OCTA can provide data on vessel density (VD) in separate layers of the macular capillary plexus. Here, we used swept-source OCTA (SS-OCTA) to examine two layers: the superficial capillary plexus (SCP) and the deep capillary plexus (DCP), and also to measure the foveal avascular zone (FAZ). These parameters have previously been used to study retinal diseases such as diabetic retinopathy and retinal vein occlusion^12–17^. However, it is still unclear what effect individual characteristics such as age, sex, IOP, and blood pressure have on OCTA measurements, because of the effect of unpreventable age-related vascular changes. Up to now, there have been only a small number of studies reporting OCTA data from at least 100 normal subjects; moreover, all these studies recruited only Western or Asian subjects and used spectral-domain OCTA, not SS-OCTA^17–19^. The differences in the macular capillary plexus in normal subjects of different ethnicities should be considered carefully^20^, and normal data obtained with SS-OCTA from Asian subjects, including Japanese subjects, are needed to perform valid worldwide comparisons.

Thus, this study sought to establish normative SS-OCTA data for Japanese subjects of a wide range of ages. We recruited 145 suitable subjects from participants in the Taiwa Study and determined macular capillary plexus density and FAZ area with SS-OCTA. We divided the subjects into subgroups by age and sex, analyzed variations in OCTA parameters between the groups, and determined the relationship of these parameters with other clinical findings, including age and blood testing results. This allowed us to determine the potential of normative SS-OCTA values to enable inter-individual and inter-group comparisons.

## Results

**Figure 1:**
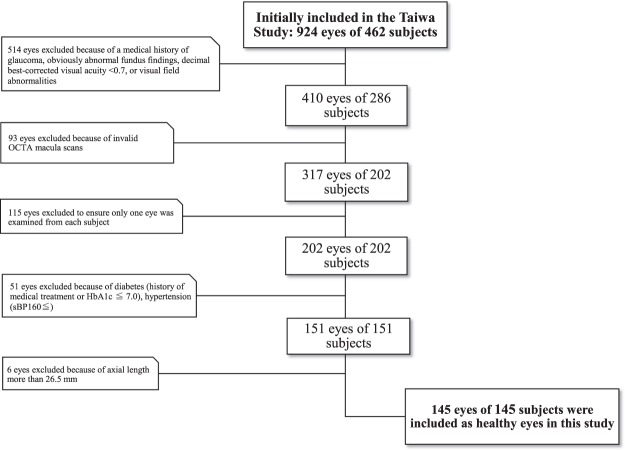
Diagram showing study design. Out of 924 eyes of 462 subjects, 145 eyes of 145 subjects were carefully selected and included as healthy eyes in this study.

The clinical characteristics of the patients are shown in Table 1. This study included 145 normal subjects (45 male/100 female; mean age: 53.3 ± 14.6 YO) (Fig. 1). The Chi-square test revealed no significant differences in sex between the age groups by decade. The one-way analysis of variance revealed significant differences in systolic blood pressure (SBP), diastolic blood pressure (DBP), triglyceride (TG), aspartate aminotransferase (AST), estimated glomerular filtration rate (eGFR), hemoglobin A1c (HbA1c), axial length, overall DCP perfusion density (PD) and FAZ area in the age groups by decade (*P* < 0.001, *P* < 0.001, *P* = 0.03, *P* = 0.02, *P* < 0.001, *P* < 0.001, *P* < 0.001, *P* < 0.01 and *P* = 0.04, respectively), but no differences in alanine aminotransferase (ALT), γ-glutamyl transpeptidase (γ-GTP), intraocular pressure (IOP), central macular thickness (CMT), overall SCP PD, parafoveal SCP PD or parafoveal DCP PD.

**Table 1:** Characteristics of eyes divided by age.

	All	30–39 YO	40–49 YO	50–59 YO	60–74 YO	*P* value
*Subjects*						
Number of eyes	145	47	13	14	71	—
Sex (male: female)	45:100	10:37	6:7	5:9	24:47	0.28^a^
Age (years)	53.3 ± 14.6	34.5 ± 2.6	44.4 ± 2.2	54.8 ± 3.3	66.0 ± 3.6	—
*General findings*						
SBP (mmHg)	121.7 ± 15.4	113.0 ± 12.7	112.4 ± 11.4	123.5 ± 15.2	128.2 ± 14.4	<0.001^b^
DBP (mmHg)	72.9 ± 10.6	68.2 ± 9.6	71.3 ± 10.7	78.6 ± 10.6	74.9 ± 10.3	<0.001^b^
TG (mg/dl)	109.5 ± 65.0	88.6 ± 63.0	98.0 ± 52.0	124.4 ± 83.7	122.6 ± 62.1	0.03^b^
AST (IU/L)	22.0 ± 6.3	20.1 ± 6.9	19.5 ± 5.0	23.3 ± 5.2	23.4 ± 6.0	0.02^b^
ALT (IU/L)	19.5 ± 9.7	19.7 ± 13.7	16.1 ± 5.6	21.4 ± 8.1	19.5 ± 7.1	0.56^b^
γ-GTP (IU/L)	27.9 ± 28.1	29.0 ± 38.7	20.0 ± 9.2	27.9 ± 18.5	28.6 ± 23.6	0.77^b^
eGFR (ml/min/1.73 m²)	82.0 ± 16.5	92.9 ± 14.4	92.4 ± 13.5	77.8 ± 14.1	73.7 ± 13.6	<0.001^b^
HbA1c(%)	5.7 ± 0.3	5.5 ± 0.2	5.5 ± 0.2	5.8 ± 0.3	5.8 ± 0.3	<0.001^b^
*Ophthalmological findings*						
Axial length (mm)	23.9 ± 1.1	24.4 ± 1.0	24.3 ± 1.2	24.1 ± 0.9	23.4 ± 0.9	<0.001^b^
IOP (mmHg)	13.8 ± 2.2	13.9 ± 2.3	14.7 ± 2.8	13.5 ± 1.2	13.5 ± 2.2	0.32^b^
CMT (μm)	295.4 ± 16.5	296.0 ± 19.1	301.4 ± 14.0	293.8 ± 13.0	294.2 ± 15.9	0.53^b^
Overall SCP PD (%)	43.6 ± 1.6	44.1 ± 1.5	43.6 ± 1.9	43.3 ± 2.2	43.3 ± 1.4	0.08^b^
Overall DCP PD (%)	44.4 ± 2.2	45.3 ± 2.0	45.0 ± 2.0	44.5 ± 2.8	43.6 ± 2.0	<0.01^b^
Parafoveal SCP PD (%)	48.2 ± 1.7	48.4 ± 1.8	48.0 ± 2.0	47.9 ± 2.4	48.2 ± 1.5	0.77^b^
Parafoveal DCP PD (%)	49.9 ± 2.6	50.7 ± 2.3	50.5 ± 2.6	49.8 ± 3.7	49.4 ± 2.5	0.07^b^
FAZ (mm²)	0.37 ± 0.1	0.33 ± 0.1	0.33 ± 0.1	0.37 ± 0.1	0.39 ± 0.1	0.04^b^

SBP = systolic blood pressure, DBP = diastolic blood pressure, TG = triglyceride, AST = aspartate aminotransferase.

ALT = alanine aminotransferase, γ-GTP = γ-glutamyl transpeptidase, eGFR = glomerular filtration rate, HbA1c = hemoglobin A1c.

IOP = intraocular pressure, CMT = central macular thickness, SCP = superficial retinal capillary plexus, PD = perfusion density, DCP = deep retinal capillary plexus, FAZ = foveal avascular zone.

^a^Chi-square test.

^b^One-way analysis of variance.

**Figure 2:**
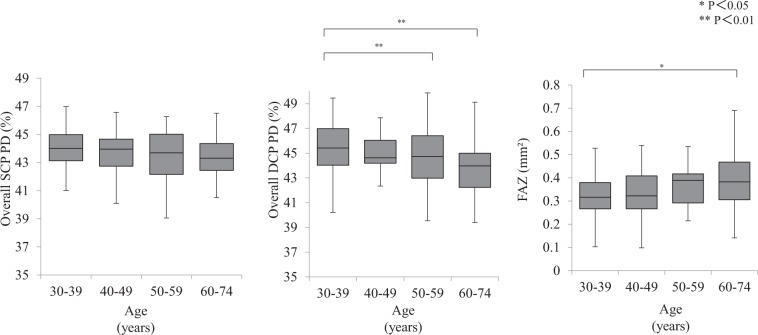
SCP and DCP PD and FAZ in the four groups classified by age. There was no significant difference in SCP PD between the four groups (left). DCP PD was lower in the 50–59 YO group and the 60–74 YO group than in the 30–39 YO group (*P* < 0.01 and *P* < 0.01, respectively; center). FAZ area in the SCP was larger in the 60–74 YO group than in the 30–39 YO group (*P* = 0.04; right).

There was no significant difference in overall SCP PD between the age groups by decade (Fig. 2 left). Overall DCP PD was lower in the 50–59 YO group and the 60–74 YO group than in the 30–39 YO group (*P* < 0.01 and *P* < 0.01, respectively; Fig. 2 center). FAZ area in the SCP was larger in the 60–74 YO group than in the 30–39 YO group (*P* = 0.04; Fig. 2 right).

A comparison of SCP and DCP PD in various areas/quadrants, based on the Early Treatment Diabetic Retinopathy Study (ETDRS), is shown in Table 2. In all subjects, DCP was higher than SCP in the overall, the parafovea, and the superior, nasal, and inferior sectors (*P* < 0.001, *P* < 0.001, *P* < 0.01, *P* < 0.001 and *P* < 0.001, respectively), while SCP was higher than DCP in the fovea (*P* < 0.001). In the 30–39 YO group, DCP was higher than SCP in the overall, the parafovea, and the superior, nasal, and inferior sectors (*P* < 0.001, *P* < 0.001, *P* < 0.001, *P* = 0.01, *P* < 0.001 and *P* < 0.001, respectively), while SCP was higher than DCP in the fovea (*P* < 0.001). In the 40–49 YO group, DCP was higher than SCP in the parafovea and the nasal and inferior sectors (*P* = 0.02, *P* < 0.01 and *P* = 0.02, respectively), while SCP was higher than DCP in the fovea (*P* = 0.02). In the 60–74 YO group, DCP was higher than SCP in the overall, the parafovea, and the nasal and inferior sectors (*P* < 0.001, *P* < 0.001, *P* < 0.001, and *P* < 0.01, respectively), while SCP was higher than DCP in the fovea (*P* < 0.001).

**Table 2:** Comparison of SCP and DCP PD in ETDRS-defined areas.

	Overall (%)	Fovea (%)	Parafovea (%)	Temporal (%)	Superior (%)	Nasal (%)	Inferior (%)
All (30–74 YO)							
SCP	43.6 ± 1.6	19.4 ± 4.8	48.2 ± 1.7	47.1 ± 2.7	50.9 ± 3.5	46.9 ± 2.8	48.8 ± 3.3
DCP	44.4 ± 3.3	15.5 ± 4.7	50.0 ± 2.6	47.4 ± 3.5	51.4 ± 4.4	49.3 ± 3.7	51.7 ± 4.3
*P* value	<0.001	<0.001	<0.001	0.49	<0.01	<0.001	<0.001
30–39 YO							
SCP	44.1 ± 1.5	21.3 ± 4.9	48.4 ± 1.8	47.3 ± 2.3	50.6 ± 3.5	47.3 ± 2.5	48.5 ± 3.5
DCP	45.3 ± 2.0	17.2 ± 4.6	50.7 ± 2.3	48.2 ± 3.4	52.4 ± 3.5	49.8 ± 3.2	52.3 ± 4.2
*P* value	<0.001	<0.001	<0.001	0.15	0.01	<0.001	<0.001
40–49 YO							
SCP	43.6 ± 1.9	20.7 ± 4.5	48.0 ± 2.1	47.4 ± 2.8	48.9 ± 3.1	47.3 ± 2.3	48.4 ± 2.1
DCP	45.0 ± 2.0	16.5 ± 4.2	50.5 ± 2.6	48.5 ± 3.2	50.5 ± 3.5	50.8 ± 2.3	52.0 ± 4.1
*P* value	0.08	0.02	0.02	0.36	0.26	<0.01	0.02
50–59 YO							
SCP	43.3 ± 2.2	19.0 ± 4.1	48.0 ± 2.4	46.1 ± 2.9	50.0 ± 3.7	47.3 ± 3.0	48.5 ± 3.5
DCP	44.5 ± 2.8	16.4 ± 5.3	49.8 ± 3.7	46.4 ± 2.9	51.6 ± 5.0	49.5 ± 5.0	51.8 ± 5.3
*P* value	0.27	0.26	0.16	0.69	0.38	0.23	0.10
60–74 YO							
SCP	43.4 ± 1.4	17.9 ± 4.5	48.2 ± 1.5	47.1 ± 3.0	50.0 ± 3.5	46.5 ± 3.0	49.2 ± 3.2
DCP	43.8 ± 2.0	14.1 ± 4.3	49.4 ± 2.5	46.8 ± 3.6	50.9 ± 4.8	48.6 ± 3.8	51.3 ± 4.3
*P* value	<0.001	<0.001	<0.001	0.56	0.19	<0.001	<0.01

SCP = superficial retinal capillary plexus, DCP = deep retinal capillary plexus, PD = perfusion density.

**Figure 3:**
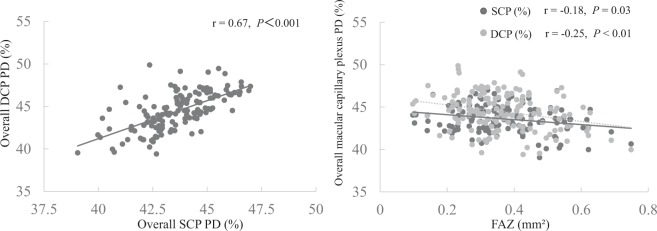
Relationship between SCP PD, DCP PD and FAZ. Left: SCP PD was correlated with DCP PD (r = 0.67, *P* < 0.001). Right: SCP and DCP PD were correlated with FAZ area (r = −0.18, *P* = 0.03 and r = −0.25, *P* < 0.01, respectively).

**Figure 4:**
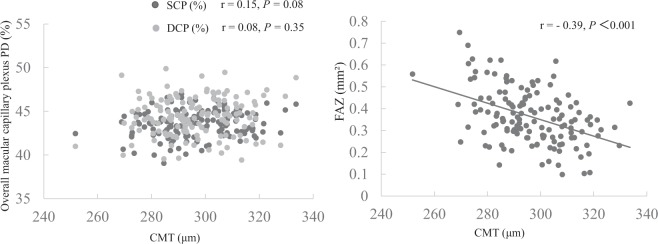
Association of CMT with SCP PD, DCP PD and FAZ. Left: SCP and DCP PD were not correlated with CMT (r = 0.15, *P* = 0.08 and r = 0.08, *P* = 0.35, respectively). Right: FAZ was correlated with CMT (r = −0.39, *P* < 0.001).

Overall SCP PD was correlated with age, eGFR, HbA1c, overall DCP PD, parafoveal SCP PD, parafoveal DCP PD and FAZ area (r = −0.17, *P* = 0.04; r = 0.17, *P* = 0.03; r = −0.18, *P* = 0.03; r = 0.67, *P* < 0.001; r = 0.87, *P* < 0.001; r = 0.59, *P* < 0.001; and r = −0.18, *P* = 0.03, respectively; Table 3 and Fig. 3 left and right), but was not correlated with SBP, DBP, TG, AST, ALT, γ-GTP, IOP or CMT (Table 3 and Fig. 4 left). Parafoveal SCP PD was correlated with overall DCP PD, parafoveal DCP PD and FAZ area (r = 0.51, *P* < 0.001; r = 0.57, *P* < 0.001; and r = 0.22, *P* = 0.01, respectively; Table 3), but was not correlated with SBP, DBP, TG, AST, ALT, γ-GTP, IOP or CMT (Table 3). Overall DCP PD was correlated with age, eGFR, parafoveal DCP PD and FAZ area (r = −0.20, *P* = 0.02; r = 0.24, *P* < 0.01; r = 0.94, *P* < 0.001; and r = −0.25, *P* < 0.01, respectively; Table 3 and Fig. 3 right), but was not correlated with SBP, DBP, TG, AST, ALT, γ-GTP, HbA1c, IOP or CMT (Table 3 and Fig. 4 left). Parafoveal DCP PD was correlated with DBP, TG and eGFR (r = −0.17, *P* = 0.04; r = −0.23, *P* = 0.01; and r = 0.18, *P* = 0.03, respectively; Table 3), but was not correlated with age, SBP, AST, ALT, γ-GTP, IOP, CMT or FAZ area (Table 3). FAZ area was correlated with age and CMT (r = 0.18, *P* = 0.03 and r = −0.39, *P* < 0.001, respectively; Table 3 and Fig. 4 right), but was not correlated with SBP, DBP, TG, AST, ALT, γ-GTP, eGFR, HbA1c or IOP (Table 3).

**Table 3:** Spearman’s rank correlation coefficient between OCTA and clinical parameters.

	Overall SCP PD		Parafoveal SCP PD		Overall DCP PD		Parafoveal DCP PD		FAZ	
	Correlation coefficient	*P* value	Correlation coefficient	*P* value	Correlation coefficient	*P* value	Correlation coefficient	*P* value	Correlation coefficient	*P* value
Age (years)	−0.17	0.04	−0.02	0.78	−0.2	0.02	−0.12	0.14	0.18	0.03
SBP (mmHg)	−0.06	0.44	−0.02	0.8	−0.15	0.07	−0.13	0.11	−0.01	0.99
DBP (mmHg)	−0.06	0.44	−0.07	0.39	−0.16	0.06	−0.17	0.04	−0.08	0.33
TG (mg/dl)	0.02	0.82	−0.01	0.95	−0.17	0.05	−0.23	0.01	−0.16	0.05
AST (IU/L)	−0.02	0.83	−0.02	0.77	−0.09	0.27	−0.07	0.41	−0.03	0.71
ALT (IU/L)	0.03	0.69	−0.01	0.97	−0.03	0.76	−0.02	0.78	−0.12	0.15
γ-GTP (IU/L)	0.05	0.51	0.04	0.62	−0.02	0.79	−0.03	0.71	−0.06	0.50
eGFR (ml/min/1.73 m²)	0.17	0.03	0.10	0.22	0.24	<0.01	0.18	0.03	−0.07	0.40
HbA1c (%)	−0.18	0.03	−0.13	0.12	−0.11	0.19	−0.08	0.35	−0.07	0.37
IOP (mmHg)	0.02	0.82	−0.02	0.82	−0.04	0.66	−0.07	0.41	−0.03	0.70
CMT (μm)	0.15	0.08	−0.01	0.89	0.08	0.35	−0.01	1.00	−0.39	<0.001
Overall SCP PD (%)	—	—	0.87	<0.001	0.67	<0.001	0.59	<0.001	−0.18	0.03
Overall DCP PD (%)	0.67	<0.001	0.51	<0.001	—	—	0.94	<0.001	−0.25	<0.01
Parafoveal SCP PD (%)	0.87	<0.001	—	—	0.51	<0.001	0.57	<0.001	0.22	<0.01
Parafoveal DCP PD (%)	0.59	<0.001	0.57	<0.001	0.94	<0.001	—	—	−0.03	0.72
FAZ (mm²)	−0.18	0.03	0.22	0.01	−0.25	<0.01	−0.03	0.72	—	—

PD = perfusion density, IOP = intraocular pressure, SBP = systolic blood pressure, DBP = diastolic blood pressure, TG = triglyceride, AST = aspartate aminotransferase, ALT = alanine aminotransferase, γ-GTP = γ-glutamyl transpeptidase, eGFR = glomerular filtration rate, HbA1c = hemoglobin A1c, CMT = central macular thickness, SCP = superficial retinal capillary plexus, DCP = deep retinal capillary plexus, FAZ = foveal avascular zone.

Separate multiple regression analyses confirmed that age was an independent factor affecting Overall DCP PD and FAZ area (β = −0.23, *P* = 0.02, Table 4 middle, and β = 0.31, *P* < 0.01, Table 4 lower, respectively) and that CMT was an independent factor affecting FAZ area (β = −0.31, *P* < 0.001, Table 4 lower).

**Table 4:** Multiple regression analysis of factors independently contributing to OCTA parameters.

Dependent	Independent	β	*P* value
Variable			
SCP PD	Age (years)	−0.14	0.16
	IOP (mmHg)	0.01	0.94
	SBP (mmHg)	0.08	0.41
	HbA1c (%)	−0.15	0.14
	CMT (μm)	0.13	0.12
DCP PD	Age (years)	−0.23	0.02
	IOP (mmHg)	−0.02	0.81
	SBP (mmHg)	−0.07	0.50
	HbA1c (%)	0.01	0.91
	CMT (μm)	0.06	0.46
FAZ	Age (years)	0.31	<0.01
	IOP (mmHg)	0.01	1.00
	SBP (mmHg)	−0.15	0.11
	HbA1c (%)	−0.16	0.08
	CMT (μm)	−0.31	<0.001

PD = perfusion density, IOP = intraocular pressure, SBP = systolic blood pressure, HbA1c = hemoglobin A1c, CMT = central macular thickness, SCP = superficial retinal capillary plexus, DCP = deep retinal capillary plexus, FAZ = foveal avascular zone.

β = standard partial regression coefficient.

Table 5 shows the characteristics of the eyes divided by age and sex. In all subjects, there were significant sex differences in DBP, TG, AST, ALT, γ-GTP, CMT and FAZ area (*P* < 0.001, *P* < 0.001, *P* < 0.001, *P* < 0.001, *P* < 0.001, *P* < 0.001 and *P* = 0.01, respectively). In younger subjects (30–59 YO), there were significant sex differences in SBP, DBP, TG, AST, ALT, γ-GTP, CMT and FAZ area (*P* < 0.01, *P* < 0.01, *P* < 0.001, *P* < 0.001, *P* < 0.001, *P* < 0.001, *P* < 0.001 and *P* < 0.01, respectively). In older subjects (60–74 YO), there were significant sex differences in TG, γ-GTP, HbA1c, axial length and CMT (*P* = 0.01, *P* < 0.001, *P* < 0.01, *P* = 0.01, and *P* < 0.01, respectively).

**Table 5:** Characteristics of eyes divided by age and sex.

	All	All subjects 30–74 YO		*P* value	30–59 YO (N = 74)		*P* value	60–74 YO (N = 71)		*P* value
		Male	Female		Male	Female		Male	Female	
*Subjects*										
Number of eyes	145	45	100	—	21	53	—	24	47	0.48^a^
Age (years)	53.3 ± 14.6	55.1 ± 13.6	51.7 ± 14.9	0.19	42.5 ± 8.6	39.1 ± 8.3	0.13	66.2 ± 4.2	65.9 ± 3.3	0.75
*General findings*										
SBP (mmHg)	121.7 ± 15.4	124.1 ± 12.9	120.2 ± 16.4	0.16	122.6 ± 13.0	111.8 ± 12.5	<0.01	125.4 ± 13.0	129.7 ± 15.0	0.23
DBP (mmHg)	72.9 ± 10.6	77.5 ± 10.4	70.6 ± 10.1	<0.001	76.7 ± 11.6	68.3 ± 9.4	<0.01	78.3 ± 9.5	73.2 ± 10.4	0.05
TG (mg/dl)	109.5 ± 65.0	145.6 ± 75.1	93.3 ± 53.3	<0.001	142.7 ± 74.1	78.9 ± 53.6	<0.001	148.1 ± 77.4	109.6 ± 48.5	0.01
AST (IU/L)	22.0 ± 6.3	24.6 ± 6.6	20.8 ± 5.9	<0.001	25.2 ± 8.2	18.8 ± 4.4	<0.001	24.1 ± 4.8	23.0 ± 6.6	0.49
ALT (IU/L)	19.5 ± 9.7	24.6 ± 11.7	17.2 ± 7.7	<0.001	27.9 ± 15.4	16.1 ± 8.0	<0.001	21.7 ± 6.3	18.4 ± 7.4	0.07
γ-GTP (IU/L)	27.9 ± 28.1	46.4 ± 43.0	19.6 ± 10.2	<0.001	51.8 ± 51.4	17.5 ± 9.4	<0.001	41.7 ± 34.5	21.9 ± 10.7	<0.001
eGFR (ml/min/1.73 m²)	82.0 ± 16.5	80.0 ± 15.0	82.9 ± 17.2	0.35	86.5 ± 15.0	91.4 ± 15.2	0.21	74.4 ± 12.7	73.3 ± 14.1	0.74
HbA1c (%)	5.7 ± 0.3	5.6 ± 0.3	5.7 ± 0.3	0.25	5.6 ± 0.3	5.5 ± 0.3	0.40	5.7 ± 0.3	5.9 ± 0.3	<0.01
*Ophthalmological findings*										
Axial length (mm)	23.9 ± 1.1	24.0 ± 0.8	23.8 ± 1.2	0.25	24.3 ± 0.7	24.4 ± 1.1	0.79	23.8 ± 0.8	23.2 ± 0.9	0.01
IOP (mmHg)	13.8 ± 2.2	13.5 ± 2.4	13.8 ± 2.1	0.37	14.0 ± 2.6	13.9 ± 2.1	0.90	13.0 ± 2.3	13.8 ± 2.1	0.20
CMT (μm)	295.4 ± 16.5	305.1 ± 13.3	291.0 ± 16.1	<0.001	308.1 ± 11.7	291.9 ± 17.0	<0.001	302.5 ± 14.3	289.9 ± 15.0	<0.01
Overall SCP PD (%)	43.6 ± 1.6	43.7 ± 1.3	43.6 ± 1.7	0.82	43.9 ± 1.1	43.8 ± 1.9	0.84	43.4 ± 1.4	43.3 ± 1.4	0.77
Foveal SCP PD (%)	19.4 ± 4.8	20.4 ± 4.7	18.9 ± 4.8	0.07	22.5 ± 4.9	20.1 ± 4.6	0.05	18.7 ± 3.8	17.5 ± 4.8	0.30
Parafoveal SCP PD (%)	48.2 ± 1.7	48.1 ± 1.6	48.3 ± 1.8	0.48	48.0 ± 1.8	48.4 ± 2.0	0.49	48.1 ± 1.4	48.2 ± 1.5	0.80
Overall DCP PD (%)	44.4 ± 2.2	44.1 ± 2.2	44.6 ± 2.2	0.19	44.9 ± 2.3	45.2 ± 2.1	0.58	43.4 ± 2.0	43.9 ± 2.0	0.28
Foveal DCP PD (%)	15.50 ± 4.7	16.7 ± 4.8	15.0 ± 4.6	0.05	18.2 ± 4.6	16.4 ± 4.6	0.13	15.3 ± 4.6	13.5 ± 4.1	0.09
Parafoveal DCP PD (%)	50.0 ± 2.6	49.3 ± 2.9	50.2 ± 2.4	0.05	50.0 ± 3.1	50.7 ± 2.5	0.30	48.7 ± 2.7	49.8 ± 2.3	0.10
FAZ (mm²)	0.37 ± 0.1	0.33 ± 0.12	0.38 ± 0.12	0.01	0.28 ± 0.12	0.36 ± 0.10	<0.01	0.37 ± 0.11	0.41 ± 0.14	0.23

SBP = systolic blood pressure, DBP = diastolic blood pressure, TG = triglyceride, AST = aspartate aminotransferase, ALT = alanine aminotransferase, γ-GTP = γ-glutamyl transpeptidase, eGFR = glomerular filtration rate, HbA1c = hemoglobin A1c, IOP = intraocular pressure, CMT = central macular thickness, SCP = superficial retinal capillary plexus, PD = perfusion density, DCP = deep retinal capillary plexus, FAZ = foveal avascular zone.

^a^Chi-square test.

The mean image quality of SS-OCTA by age group was as follows, for the 30–39 YO, 40–49 YO, 50–59 YO and 60–74 YO patients, respectively: 63.1 ± 6.9, 64.9 ± 6.1, 66.4 ± 5.9, 62.0 ± 6.6 and 61.4 ± 7.1. There were no significant differences between most of these groups, with the exception of the 30–39 YO and 60–74 YO groups (*P* = 0.02).

In all included subjects, intra-observer reproducibility of for FAZ area was as follows: intraclass correlation coefficient (ICC) = 0.96 and coefficient of variation (COV) = 5.8%, and inter-observer reproducibility for FAZ area was as follows: ICC = 0.97 and COV = 3.7%.

## Discussion

This study establishes a normative database for OCTA measurements of the macular capillary plexus, which promises to allow inter-subject and inter-group comparisons, thereby improving the diagnosis of retinal diseases and glaucoma. We used SS-OCTA to determine PD in the SCP and DCP and FAZ area and calculated the relationship between these results and general findings in healthy Japanese subjects ranging widely in age, all of whom were recruited as part of the Taiwa Study. Our main findings were that SCP and DCP PD were negatively correlated with age, while FAZ area was positively correlated with age. Moreover, SCP and DCP PD showed a positive correlation with each other, while FAZ area showed negative correlations with SCP PD, DCP PD and CMT. FAZ was larger and CMT was lower in the female subjects than the male subjects. SCP and DCP PD were both positively correlated with eGFR. Multiple regression analysis confirmed that age was an independent factor affecting DCP PD and FAZ and that CMT was also an independent factor affecting FAZ area.

### Comparison with previous normative data for SCP and DCP PD

There have been only a few studies discussing OCTA-measured VD, all of which used circular regions of interest (ROI) in the normal macula^17,18,21–23^. In one such previous study, PD in the parafoveal SCP and DCP was reported to be 46.0% and 51.5%, respectively^22^, similar to our findings of 48.2% and 50.0%. However, the same study reported that PD in the foveal SCP and DCP was 31.9% and 27.5%, respectively^22^, which is much higher than our findings. This might be explained by the fact that the previous study defined the foveal region as a central, 1.2-mm circle comprising 120 pixels, in contrast with the current study, which used a 1.0-mm circle. Circular ROIs for VD are the most suitable to compare VD in different sectors of the macula, considering the anatomy of the macular vascularity and the fact that the distance from the foveal center is fixed. The current study, which also used a circular ROI, adds support to existing findings that DCP PD is higher than SCP PD in the parafovea, but, inversely, is lower in the fovea^21,22^. Higher DCP PD in the parafovea may be explained by the composition of the SCP and DCP. Specifically, the SCP includes transverse capillaries and the DCP includes the homogenous capillary vortex^23,24^.

Furthermore, interestingly, our study found no significant differences in SCP and DCP PD in the temporal sector, regardless of age. This might be because DCP PD in this sector is relatively low, possibly due to the specific anatomical vascularity of the temporal raphe^25^, a horizontal boundary separating the superior and inferior retinal nerve fiber bundles in the temporal retina, i.e., the watershed zones. Our study also showed a positive correlation between SCP and DCP PD in the overall area. Considered together, these findings show that in normal eyes, there is a close association between SCP and DCP PD, and that SCP PD is higher in the foveal area, while DCP PD is higher in the parafoveal area.

### Aging

Generally, the health of the human vascular system is closely associated with age. The current study demonstrated that SCP and DCP PD were negatively correlated with age, while SCP FAZ area was positively correlated with age. This is consistent with previous studies showing similar relationships between changes in the macular capillary plexus and age^17,18,22,23^. These age-related changes have been reported to be caused by occlusion and atrophy of the retinal capillaries^26^. However, in contrast to DCP PD, SCP PD was similar in the four groups in the current study, and furthermore, a multivariate analysis confirmed that only DCP was closely associated with age (with a per-year decrease of 0.23%). Although a previous study that investigated human donor eyes with confocal microscopy found no age-dependent changes in retinal capillary density^27^, previous studies using methods more similar to ours found annual decreases in the macular capillary plexus and annual increases in FAZ^17,18^. Thus, we speculate that DCP PD decreases and SCP FAZ increases with age, probably because of reductions in oxygen and nutrient demand, in the normal population.

We observed only weak tendencies in the data from the middle-aged subjects (i.e., the 40–49 YO and 50–59 YO groups), probably due to the small numbers of subjects in these groups. These small group sizes were possibly due to lifestyle factors, which do not allow middle-aged individuals to attend annual medical checks.

### Sex

Male sex has previously been reported to be associated only with increased SCP PD^28^. In our study, though there were sex differences in some background characteristics, including blood pressure and hepatic and biliary enzymes (which also differed somewhat in the younger and older groups), our results are compatible with previous reports that there were no sex differences in PD^21,22,29,30^. Furthermore, the central subfield and foveal and parafoveal thickness have been reported to be lower in women than men^20,31^. Our finding that CMT was lower in the female than the male subjects confirms that this tendency is present regardless of age. Women have also been reported to have a larger superficial and deep FAZ^32^. Similarly, our results showed that, in all subjects and in the younger subjects, FAZ was larger in the female than the male subjects. There were no sex differences in FAZ among the older subjects, possibly because axial length was longer in the male than in the female subjects. Considered together with past findings, we speculate that there are no sex differences in PD, although there are in CMT and FAZ.

### FAZ

We found that FAZ area increased with age^18,33^. Enlarged FAZ (a novel OCTA parameter) has also previously been reported to be associated with various ocular diseases, including diabetic retinopathy and retinal vein occlusion^34,35^. Since the border of the FAZ is more clearly visible at the level of the SCP than the DCP^36,37^, many investigators have focused on SCP FAZ^37–41^. Reproducibility has also been reported to be relatively low in measurements of DCP FAZ. Intra- and inter-observer agreement are both better in SCP FAZ than in DCP FAZ^42^. Furthermore, studies of the macular capillary plexus that use manufacturer-recommended default settings might be biased^43^, and we speculate that measurements of DCP FAZ may be especially unreliable because of stronger errors in segmentation of the retinal layers when the default settings are used.

We found that FAZ in the SCP measured 0.37 mm^2^, which is comparable to previous results for FAZ (0.30~0.35 mm^2^), measured both with OCTA and with other approaches such as fluorescein angiography and scanning laser ophthalmoscopy^37–41^. We also found that FAZ area was negatively correlated with SCP and DCP PD, which is also consistent with previous findings^21^. FAZ has previously been shown to be larger in eyes with a deeper and broader foveal pit^44^, while a small FAZ has been shown to be a historic mark of prematurity^45^. In pediatrics, a larger FAZ was significantly associated with older age and with reduced foveal macular thickness^28^. Furthermore, our multivariate analysis showed that FAZ was negatively correlated with CMT, again agreeing with previous results^21,36,46^. Thus, current and previous findings indicate that SCP FAZ area increases significantly as CMT decreases. A positive correlation between inner retinal thickness and parafoveal PD has also been reported in healthy subjects^47^. Therefore, considering all results together, we consider that parafoveal vascularity is most likely closely associated with macular thickness.

### Associations between OCTA and general findings

The current study is the first to evaluate the association between SS-OCTA parameters and general findings, including renal function. Though renal function can already be evaluated based on eGFR, we found, interestingly, that overall SCP PD, overall DCP PD, and parafoveal DCP PD were positively correlated with eGFR. We included 6 different blood tests, but eGFR was the only one associated with both types of PD. This may be explained by past reports showing a close relationship between renal function and ocular circulation^48–50^. In older subjects (more than 60 YO), eGFR has been reported to be independently related to central retinal arteriolar equivalent^49^. The progression of chronic kidney disease has also been reported to be significantly and independently associated with decreased retinal blood flow, measured by a laser Doppler velocimetry system, in early-phase diabetic retinopathy^50^. Furthermore, in a study using static and continuous retinal vessel responses to three cycles of flickering light, eGFR was found to be linked to arterial reaction time, arterial maximum dilatation and the dilatation amplitude responses in diabetes and cardiovascular disease^48^. Thus, since there might be a close relationship between renal function and ocular circulation, renal function must be considered when interpreting retinal circulation.

### Limitations and advantages

The current study was somewhat limited by its cross-sectional design. Additionally, we did not use a third, intermediate retinal capillary plexus, but only SCP and DCP. The number of middle-aged subjects was also relatively small, because of the restricted study design. Another possible limitation is that we used age, but not eGFR, in our multiple regression analyses. We made this choice because both the present and previous reports have shown that age and eGFR are closely correlated (current study: r = −0.56, *P* < 0.001)^51–53^.

This study also had many advantages, including a sample size that was large overall, included only one eye from each subject, included only a single ethnicity, and had a wide age range. Reproducibility in the measurements of FAZ was also very high (intra-observer and inter-observer reproducibility: both ICC > 0.95 and both COV < 6%). The study also used strict inclusion criteria to define healthy subjects, confirmed by blood tests and the decisions of medical doctors. Furthermore, to reduce axial length-related data bias, eyes with an axial length greater than 26.5 mm were excluded from this study. Mean image quality was more than 60 in all groups, suggesting that our analysis had good quality. There were no significant differences between most of the groups (with the exception of the youngest and oldest groups) likely due to senile cataract.

## Conclusion

In conclusion, we found that macular capillary plexus parameters, measured with SS-OCTA, were closely associated with age and general findings, and found that SS-OCTA was an excellent way of obtaining data on retinal vascularity in separate layers of the macular capillary plexus. Our key findings were that SCP and DCP PD were negatively correlated with age, while FAZ area was positively correlated with age. Additionally, SCP and DCP PD showed a positive correlation with each other. FAZ area showed a negative correlation with SCP PD, DCP PD and CMT, and FAZ was larger, while CMT was lower, in the female subjects than the male subjects. Finally, SCP and DCP PD were both positively correlated with eGFR. Thus, our findings indicate that macular capillary vascularity decreases with age, in association with declining renal function, in normal subjects. Moreover, the relationship between FAZ area and CMT suggests that changes in age-related macular thickness occur in close association with changes in capillary vasculature. We believe that the current results, obtained from normal subjects, should be helpful in improving clinical diagnoses and should allow the comparison of macular capillary plexus parameters obtained with SS-OCTA.

## Methods

### Setting and design

This was an institutional, cross-sectional case series.

### Patients

Recruited from the Taiwa Study; 924 eyes of 462 subjects. All subjects attended an annual medical check in the town of Taiwa, northern Japan, on the 26^th^ and 29^th^ of May or the 3^rd^ and 4^th^ of June in 2017. Subjects were excluded if they had a medical history of glaucoma, obviously abnormal fundus findings, decimal best-corrected visual acuity < 0.7, visual field abnormalities, axial length more than 26.5 mm, diabetes (history of medical treatment or HbA1c ≧ 7.0), or hypertension (SBP ≧ 160). Finally, this study included 145 eyes of 145 healthy Japanese subjects who underwent 3 × 3 mm OCTA scans of the macula as part of the Taiwa Study (Fig. 1).

The institutional review board of the Tohoku University Graduate School of Medicine approved this study (No. 2017-1-254). Informed consent was obtained from each patient for his or her participation in the research, and the research was conducted according to the provisions of the Declaration of Helsinki, 1995 (as revised in Edinburgh, 2000).

### Main outcome measure

Standard statistical techniques were used to determine associations between clinical findings, including age, IOP, axial length, SBP, DBP, CMT, SCP PD, DCP PD, and FAZ area in the SCP.

### Measurement of physical and ophthalmological findings

SBP and DBP were measured after the patients had rested in a sitting position for 10 min. Measurements were made in the left brachial artery at the height of the heart with an automated blood pressure monitor (HEM-759E, Omron Corporation, Kyoto, Japan). Levels of TG, AST, ALT, γ-GTP, and HbA1c, as well the eGFR, were measured with automated standardized laboratory techniques. Ophthalmological examinations included fundus photography (SS-OCT Angio, Topcon Corporation, Tokyo, Japan), visual acuity, axial length (IOL-Master, Carl Zeiss Meditec, Oberkochen, Germany) and IOP (auto-refractometry).

### Measurement of macular capillary plexus and thickness

Subjects underwent 3 × 3 mm OCTA scans (SS-OCT Angio, Topcon Corporation, Tokyo, Japan) of the macula. We defined the PD as the total area of perfused vasculature per unit area in a region of measurement, in conformity with a previous paper^54^. PD was calculated in a fovea-centered 2.5-mm diameter circle (whole retina). PD was measured in an inner, 1.0-mm diameter ring around the foveal center, (i.e., the fovea), a larger, 2.5-mm diameter ring (i.e., the parafovea), and in the four quadrants (i.e., temporal, superior, nasal and inferior) PD calculation was based on the ETDRS. We measured the PD in the SCP and DCP, and FAZ area in the SCP, and determined the relationship between these parameters and other clinical findings, including age and the results of blood testing. We use customized third-party software (IMAGEnet6 version 1.23) to remove the large vessels. The resulting OCTA images, used to calculate the vessel density, were not skeletonized, but only binarized. We also determined the correlation between CMT and these parameters. To determine CMT, 7 × 7 mm OCT cube scans centered on the fovea were made. CMT was automatically calculated by averaging the retinal thickness of the macula within 1 mm of the fovea. All included SS-OCT data had a minimum image quality > 35; images were excluded if automatic layer segmentation failed or we judged an image as having significant artifacts.

Automatic analysis software (Topcon Corporation, Tokyo, Japan) was used to set a dividing line 15.6 μm below the boundary between the inner plexiform layer and inner nuclear layer in the macula (i.e., below the SCP and above the DCP). This novel system automatically identified the foveal center and measured SCP and DCP within a 2.5-mm circle. FAZ area in the SCP was calculated manually with ImageJ software. We then determined the statistical associations between these measurements.

OCTA data can be corrected for axial length, but the effects of this correction are still open to debate. Furthermore, we consider that correction might not be the best choice for evaluating the capillaries of the macula. Therefore, in this study, we decided to use uncorrected OCTA data

### Reproducibilty

The repeatability and reproducibility of the VD results obtained with this software were not investigated. However, the ICC and the COV were calculated to determine the intra- and inter-observer reproducibility of the manually determined data for FAZ area. The intra-observer reproducibility was determined by analyzing results from one observer, who performed the measurements twice. The inter-observer reproducibility was determined by analyzing results from two observers.

### Statistical analyses

The data are presented as mean ± standard error of the mean. The subjects were divided into age groups by decade (i.e., 30–39 YO, 40–49 YO, 50–59 YO and 60–69 YO); the subjects were also divided into younger and older groups, comprising those 30–59 YO and 60–69 YO, respectively. The Chi-square test was used to evaluate differences in sex between the age groups. A one-way analysis of variance was used to evaluate differences in SBP, DBP, TG, AST, ALT, γ-GTP, eGFR, HbA1c, axial length, IOP, CMT, SCP PD, DCP PD and FAZ area in the age groups. Spearman’s rank correlation test was used to estimate the relationships of SCP PD, DCP PD and FAZ area to SBP, DBP, TG, AST, ALT, γ-GTP, eGFR, HbA1c, axial length, IOP, CMT, SCP PD, DCP PD and FAZ. The mean image quality of SS-OCTA by age group was compared with the Tukey–Kramer test. Separate multiple linear regression analyses were performed to analyze each independent variable potentially affecting SCP PD, DCP PD and FAZ. All statistical analyses were performed with JMP software (Pro version 10.0.2, SAS Institute Japan Inc., Tokyo, Japan). Differences were considered significant at *P* < 0.05.
